# Intra- and inter‐observer reliability of implant positioning evaluation on a CT-based three‐dimensional postoperative matching system for total knee arthroplasty

**DOI:** 10.1186/s12891-021-04228-2

**Published:** 2021-04-17

**Authors:** Shotaro Watanabe, Ryuichiro Akagi, Yuki Shiko, Yoshimasa Ono, Yohei Kawasaki, Toshihiro Ohdera, Seiji Ohtori, Takahisa Sasho

**Affiliations:** 1 Department of Orthopaedic Surgery, Graduate School of Medicine, Development of Orthopaedic Surgery, Chiba University, 1-8-1 Inohana, Chuo-ku, Chiba, Japan; 2 Center for Preventive Medical Sciences, Chiba University, 1-8-1 Inohana, Chuo-ku, Chiba, Japan; 3 Biostatistics Section, Clinical Research Center, Chiba University Hospital, 1-8-1 Inohana, Chuo-ku, Chiba, Japan; 4 Fukuoka Orthopaedic Hospital, 2-10-50 Yanagouchi, Minami-ward, Fukuoka-city, Fukuoka Japan

**Keywords:** Total knee arthroplasty, Implant position, Postoperative evaluation, 3D-CT, Intra‐observer reliability, Inter‐observer reliability

## Abstract

**Background:**

The evaluation of postoperative total knee arthroplasty (TKA) alignment mainly relies on measurement data obtained from plain radiographs. The aim of this retrospective observational study was to document the intra- and inter-observer reliability in assessment of TKA component positioning after surgery using a three-dimensional (3D) computed tomography (CT) image matching system.

**Methods:**

Fourteen knees from 14 patients who received primary TKA were included, and images were analyzed by blinded readers not associated with the surgeries. The examiner digitized the reference points according to defined landmarks, and the designated size component was superimposed to the 3D reconstructed CT model for measurement. In addition to the evaluation of implant position against the coronal and sagittal lower limb mechanical axes that were defined based on bony landmarks, implant position against axes connecting implant-based reference points that are easier to indicate was evaluated.

**Results:**

The overall intra- and inter-observer reliabilities determined by the intraclass correlation coefficients (ICC) of the implant alignment measurement for both femoral and tibial components were good (ICC > 0.60), except in the direction of femoral flexion and extension, for both mechanical and implant-based axes. The difference between implant alignment measurements according to the traditional mechanical axis and the implant-based axis ranged between means of 0.08^o^ and 1.70^o^ and were statistically significantly different.

**Conclusions:**

The postoperative evaluation of implant position in the coronal and sagittal planes using 3D-CT image matching is reliable and has good reproducibility except for the sagittal alignment assessment of the femoral component. The measured implant position according to the traditional mechanical axis and the implant-based axis were slightly but significantly different.

## Background

Malposition of the prosthesis can negatively affect clinical outcomes and extended survivorship after total knee arthroplasty (TKA) [1, 2]. The majority of cases can achieve successful results in accurately positioning the prosthesis by traditional surgical techniques using intra- and extra-medullary guides. However, some do not necessarily result in enough accuracy, with errors occurring for multiple reasons such as the patients’ anatomical variation, the surgical procedure, or the surgeon’s determination of anatomical landmarks [3–5]. As many as 20–30 % of patients may deviate from preoperative planning by more than three degrees of error [6–8]. Many ancillary techniques and devices have been proposed to align the prosthesis accurately and reduce outliers. Among them, computer-assisted intraoperative navigation has gained more attention during the last decade, with many authors describing significant improvement in implant positioning [7, 8].

The limitation of previous reports is that the reproducibility of the evaluation method for postoperative alignment often has not been validated [9, 10]. It is essential to establish a reproducible and accurate method to evaluate the position of the implant after surgery in order to assess whether the implant has been placed correctly. The majority of studies rely on measurement data obtained from plain radiographs [1, 11, 12], and the correlation of outcomes to postoperative alignment may be affected by the type of radiographs used to measure alignment [1]. Recent studies report greater accuracy by using three-dimensional (3D) computed tomography (CT)-based imaging techniques compared to conventional radiography-based methods for preoperative planning of TKA [13]. Some of these 3D-CT techniques can be applied to postoperative CT images as well to evaluate implant alignment after surgery by superimposing the computer-aided-design (CAD) model onto the implanted prosthesis on the postoperative CT. Yoshino et al. reported favorable intra- and inter-observer reliabilities of this technique in all planes except for the sagittal position of the femoral component [14]. However, there is a potential concern of measurement error in this evaluation technique due to difficulties of matching the CAD model on CT images and determining bony landmarks to define the reference axis, resulting from the halation of the implant.

Traditionally, the mechanical axis of the lower limb has been defined as the axis connecting the center of the hip and the center of knee joint for the femur, and the center of the knee and ankle joints for the tibia [15]. However, as some of the reference points are difficult to identify on CT scans obtained after TKA due to halation caused by the implant, we measured another axis, in addition to the traditional mechanical axis, that uses the center of the femoral and tibial components as reference points to define the axes. These two axes, the traditional mechanical axis and the implant-based axis, are similar but not equal. The postoperative measurement and evaluation of implant alignment accuracy may be slightly different between these two axes.

In this observational study, we aimed to (1) measure the intra-observer and inter-observer reliabilities in evaluating TKA component positioning after surgery by the 3D-CT evaluation system, and (2) analyze the difference of implant position measurements between the traditional mechanical axis and the implant-based axis. Our hypotheses were that the postoperative evaluation using 3D-CT image matching is reliable with good reproducibility and that there would be a substantial difference in the measured postoperative implant alignment between the traditional mechanical axis and the implant-based axis.

## Methods

This study was conducted by evaluating a series of anonymized images collected after TKA in a multicenter prospective study (NCT number: NCT03227692, Japan Registry of Clinical Trials Trial ID: jRCTs032180377). The authors were requested to take part as an independent evaluation group for the multi-center study. The current study was conducted prior to the multi-center study to validate the measurement method. The research protocol for the study was approved by the institutional review board, and written informed consent was obtained from each patient at the time of inclusion in the study. The study was conducted following the guidelines for reporting reliability and agreement studies [16].

### Patients and surgical technique

Patients who received primary TKA between August 2017 and September 2019 and participated in the multicenter study were included in this study. Images of the first sequential 14 knees from 14 patients were analyzed by blinded readers not associated with the surgeries. Patient demographic data, including sex and age, were not provided to the readers since they were considered unnecessary for this study. The Persona™ Knee (Zimmer Biomet, Warsaw, Indiana, USA) was used in all cases with cement fixation. The surgeons decided whether to use posterior-stabilized (PS) or cruciate-retaining (CR) types of component, according to their preferences.

### Image acquisition

All patients underwent a CT scan six months after surgery. Images were acquired sequentially from the hip joint to the ankle joint, with a slice thickness of 1.25 millimeters. Full limb CT was necessary for the analysis due to software specifications. The autoexposure control system was utilized to minimize radiation exposure [17]. Images were exported as anonymized digital imaging and communications in medicine (DICOM) data format and sent to the independent evaluation organization.

### Image reconstruction and measurement

Image data were imported into a CT-based 3D preoperative planning and postoperative evaluation system (ZedView™, LEXI Co., Ltd., Tokyo Japan) for reconstruction and evaluation. Three orthopaedic surgeons with more than six years of experience in knee arthroplasty and reading of musculoskeletal images independently evaluated the images, blinded to each other’s measurements. All examiners were blinded to the surgical technique, but information on the implant size and type of prosthesis (PS or CR) was provided. Multiplanar reconstruction images in the sagittal and coronal planes were automatically created from the axial plane CT images. The examiner digitized the reference points according to defined landmarks [13] (Table 1), and a 3D reconstruction model was created. The tip of the medial epicondyle was chosen for the reference point at the distal femur since the groove of the medial epicondyle was challenging to define on the CT images in some cases. After the halation of the implant was minimized using the software, the CAD model of the designated size component was superimposed onto the CT scan image by matching the outer rim, peg of the femoral component, and stem on the tibial component.

**Table 1:** Bony landmarks as reference points for 3D-CT digitizing to define the traditional mechanical axis

Reference point	Description
Center of the femoral head	Four points were set on the surface of the femoral head (three in the sagittal or coronal plane, and another point in the axial plane). A ball that contacts all four points was automatically created to approximate the femoral head. The center of the ball was defined as the center of the femoral head.
Medial/lateral epicondyle of the femur	The tip of the medial/lateral epicondyle was marked on the axial plane, by referring to the coronal plane at the same time.
Proximal/distal center of the tibia	Three points were set in the axial plane inside the medullary canal of the tibia at the proximal/distal one-third of the tibia. The center of the circle that contacted all three points was defined as the center of the proximal/distal tibia.

**Fig. 1:**
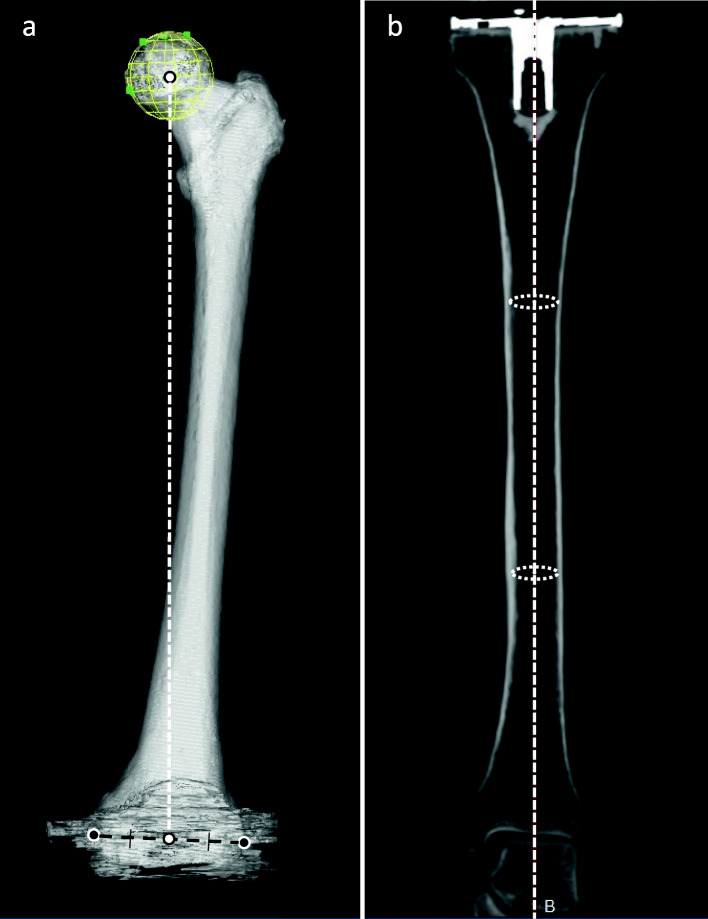
Reference points of the traditional mechanical axis . **a**: Three-dimensional image of the femur from the anterior aspect. The traditional mechanical axis of the femur was defined as the line connecting the white dots, which indicates the center of the femoral head and the center of the medial and lateral epicondyles (black dots). **b**: Multi-planar reconstruction coronal image of the tibia. Traditional mechanical alignment of the tibia was defined as the axis connecting the center of the circles drawn inside the upper and lower one-third of the medullary canal of the tibia

Measurements were performed on two different reference axes. In addition to the evaluation of implant position against the traditional lower limb mechanical axis [18] in the coronal and sagittal planes, implant position against the implant-based axis was evaluated. Traditional lower limb mechanical alignment was defined as the axis connecting the center of the femoral head and the center between the medial and lateral epicondyles of the femur (Fig. 1a), and the axis connecting the center of the upper and lower one-third of the medullary canal of the tibia (Fig. 1b). As mentioned above, the tip of the medial epicondyle was registered at the distal femur. We did not assess rotational alignment in this study as the original research design of the multi-center study did not require this. and the difference between the center of the anatomical and the surgical trans-epicondylar axis was considered negligible in defining the coronal and sagittal femoral axis.

**Fig. 2:**
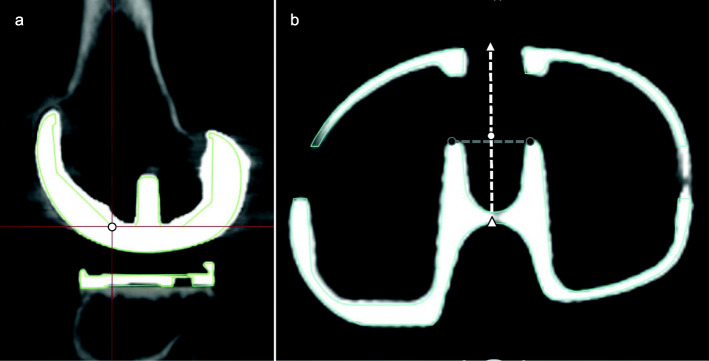
Reference points of the implant-based axis. **a**: Multi-planar sagittal reconstruction image of the femoral component. The mediolateral center at the distal end of the anterior chamfer was defined as the center of the femoral component. **b**: Multi-planar reconstruction axial image of the tibial tray. A perpendicular bisector of a line segment connecting the anterior end of the dovetails (black dots) and the center of the anteroposterior edges of the tibial tray (white triangles) was defined as the center of the tibial tray (white dot)

The implant-based femoral axis was defined as the axis connecting the center of the femoral head and the center of the femoral component at the base of the anterior chamfer (Fig. 2a). The implant-based tibial axis was defined as the axis connecting the center of the tibial tray and the center of the ankle joint. To define the center of the tibial tray, the center of the transverse diameter of the tibial tray was marked as the center of the anterior end of the dovetail. Next, in the sagittal plane that passes through the center of the transverse point, the center of the anteroposterior diameter of the tibial tray was defined as the center of the tibial tray (Fig. 2b). The center of the ankle joint was defined as the center of the line connecting the center of the articular surface of the medial malleolus and lateral malleolus at the level of the tibial plafond. This point corresponded to the center of the line connecting the medial and lateral peaks of the talar dome.

**Fig. 3:**
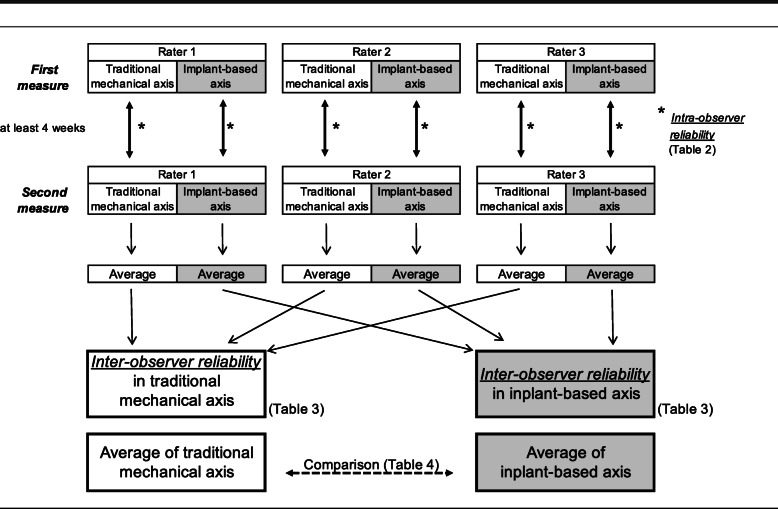
Experimental design and analyses. Each examiner independently performed the measurement of sagittal and coronal alignments twice for all images, with at least four weeks between measurements. The intra-observer ICC was calculated for each of the three observers, and the inter-observer ICC was calculated using the average of the two measurements of each observer. Implant alignment measurements were compared between the traditional mechanical axis and the implant-based axis

The sagittal and coronal inclination of both femoral and tibial components were measured according to each axis. The angles were recorded in positive values when the coronal plane inclination was in varus. In the sagittal plane, the values were recorded positive when the femoral component was in a flexed position, or the tibial component had a posterior slope. Each examiner independently performed the measurement twice for all images, with at least four weeks between measurements. The whole process, including the digitizing of the reference point, was repeated from the beginning of the second measurement (Fig. 3).

### Sample size and statistical analysis

The intra-observer intraclass correlation coefficient (ICC) was calculated for each of the three observers, and the inter-observer ICC was calculated using the average of the two measurements of each observer (Fig. 3). We defined the ICC (= 0.80) and the width of the confidence interval (CI = 0.40) for inter-observer reliability in assessment of the femoral valgus angle on a 3D-CT system from a previous report [13]. We also considered the ICC setting using three evaluators based on past literature [19]. These conditions were used to determine the number of cases for this study. Based on these assumptions, the required sample size was 14 in this study [19].

The ICCs with a 95 % confidence interval (95 % CI) were calculated for intra- and inter-observer reliability in assessment of TKA component alignment after surgery using the 3D-CT system. An ICC value of 1 was considered perfect, > 0.80 very good, > 0.60 good, and > 0.40 moderate reliability [20]. Implant alignment measurements were compared using paired t-tests between two groups (the traditional mechanical axis and the implant-based axis) (Fig. 3). A two-sided test result of p < 0.05 was considered statistically significant. All results were analyzed using SAS version 9.4 for Windows (SAS Institute, Cary, NC, USA).

## Results

### Intra‐observer reliability

The ICCs representing intra-observer reliabilities for measurement of the position of the components on 3D-CT images are presented in Table 2. The overall intra-observer ICCs of the implant alignment measurement for components using both the traditional mechanical axis and the implant-based axis were generally very good. The ICC for intra-observer reliability by one of the raters was moderate for the measurement in flexion-extension of the femoral component for both traditional and implant-based axes.

**Table 2:** The ICCs for intra-observer reliability

Index	Component	Alignment	Rater	ICC	95 %CI
Traditional mechanical axis	Femoral	Varus - Valgus	1	0.92	0.78–0.97
			2	0.98	0.94–0.99
			3	0.87	0.66–0.95
		Flexion - Extension	1	0.49	0.00–0.79
			2	0.80	0.51–0.93
			3	0.80	0.51–0.93
	Tibial	Varus - Valgus	1	0.78	0.47–0.92
			2	0.88	0.69–0.96
			3	0.93	0.81–0.98
		Anterior - Posterior Slope	1	0.91	0.76–0.97
			2	0.94	0.83–0.98
			3	0.91	0.76–0.97
Implant-based axis	Femoral	Varus - Valgus	1	0.93	0.81–0.98
			2	0.97	0.92–0.99
			3	0.85	0.62–0.95
		Flexion - Extension	1	0.50	0.02–0.79
			2	0.72	0.36–0.89
			3	0.75	0.41–0.91
	Tibial	Varus - Valgus	1	0.86	0.64–0.95
			2	0.86	0.64–0.95
			3	0.94	0.83–0.98
		Anterior - Posterior Slope	1	0.93	0.81–0.98
			2	0.95	0.86–0.98
			3	0.89	0.71–0.96

*CI* confidence interval

### Inter‐observer reliability

The ICCs representing inter-observer reliability for measurement of the position of the components on 3D-CT images are presented in Table 3. The overall ICC of the implant alignment measurement for both femoral and tibial components was very good for both the traditional mechanical axis and the implant-based axis.

**Table 3:** The ICCs for inter-observer reliability

Index	Component	Alignment	ICC	95 %CI
Traditional mechanical axis	Femoral	Varus – Valgus	0.97	0.94–0.99
		Flexion - Extension	0.89	0.78–0.95
	Tibial	Varus - Valgus	0.90	0.80–0.95
		Anterior - Posterior Slope	0.94	0.88–0.97
Implant-based axis	Femoral	Varus - Valgus	0.96	0.92–0.98
		Flexion - Extension	0.82	0.65–0.91
	Tibial	Varus - Valgus	0.74	0.52–0.87
		Anterior - Posterior Slope	0.96	0.92–0.98

*CI* confidence interval

### Comparison of implant positioning measurements for the traditional mechanical axis and the implant‐based axis

The results of implant alignment measurements according to the traditional mechanical axis and implant-based axis are presented in Table 4. The gap between the two values varied from 0.08° to 1.70° on average, and all measurements reached statistical difference. A larger difference in measured alignment was observed in the sagittal plane compared to the coronal plane for the femoral component positioning, while the difference was larger in the coronal plane than the sagittal plane for the tibial component position.

**Table 4:** Differences of components’ alignment between measurements using the traditional mechanical axis or the implant-based axis

Component	Plane	Traditional mechanical axis (degrees)	Implant-based axis (degrees)	Difference		*p* value
				**Mean**	**Range**	
Femoral	Coronal^a^	-0.28	-0.19	0.08	-0.55, 0.27	< 0.001
	Sagittal^b^	1.42	3.11	1.70	-2.44, -0.36	< 0.001
Tibial	Coronal^a^	1.03	1.60	0.56	-2.11, 1.04	< 0.001
	Sagittal^c^	3.18	3.38	0.19	-1.64, 1.22,	0.0055

^a^Positive values for varus alignment.

^b^Positive values for flexion alignment.

^c^Positive values for posterior slope

## Discussion

Our results confirmed the good reproducibility of the 3D-CT based postoperative evaluation system in measuring TKA component positioning, although the sagittal alignment assessment of the femoral component was less reliable. We were able to demonstrate a small but significant difference between measurements based on the traditional mechanical axis and those based on the implant-based axis.

Appropriate limb alignment to obtain satisfactory outcomes after TKA has not been defined clearly despite debate for many years [8, 21]. Nevertheless, regardless of the underlying theory, surgeons try to position the implant in the manner they believe is “optimal” as the accuracy of implant positioning is essential to obtain positive surgical outcomes. Discussion continues about the optimal postoperative measurement of alignment. Evaluation methods applied in previous studies to assess TKA positioning after surgery typically include plain anteroposterior and lateral radiographs or two-dimensional CT images [15, 21–23]. Many studies do not report the accuracy of the evaluation technique itself [24], yet the accuracy and reproducibility of the measuring technique are critical factors that may bias the results. Plain radiographs are used to assess limb alignment in the coronal plane but may not be accurate enough for assessment of tibial and femoral TKA position due to multiple factors such as limb rotation and knee flexion contracture [25]. 3D-CT-based imaging techniques can be expected to provide better accuracy in the evaluation. Franceschi et al. evaluated the reproduction of 3D-CT preoperative planning by comparing it to the postoperative CT image by superimposing pre- and postoperative CT scans and reported good reproducibility [26].

A limited number of studies report the use of 3D-CT analysis for component position measurements after TKA [27, 28]. These reports used the method reported by Hirschmann et al., [29] which has a high inter- and intra-observer reliability for component position evaluation [25, 30]. However, the method to determine the component position was not described in detail. The accuracy and reproducibility of evaluation could be influenced not only by the imaging methods [21], but also by how the reference points and the axis were determined by the raters. In the present study, we measured the intra- and inter-observer reliability in evaluating TKA component positioning after surgery by a 3D-CT evaluation system. The system we used in this study is one of the most common validated methods to perform preoperative planning [13], and also can be used for postoperative evaluation. Yoshino et al. evaluated the same postoperative implant alignment assessment method as our current study, using the 3D-CT image matching technique, and reported good intra- and inter-observer reliability except for the assessment of sagittal alignment of the femoral component [14]. Our results confirmed the good reproducibility of the system, both for intra- and inter-observer reliability, demonstrating its usefulness in postoperative assessment.

**Fig. 4:**
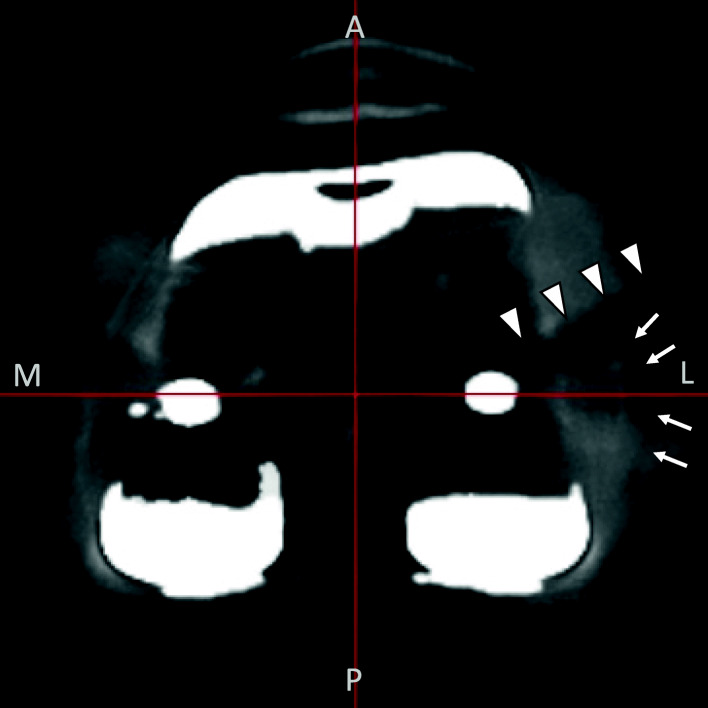
A case in which the anatomical landmarks are difficult to identify because of implant halation The lateral epicondyle (arrow) is obscured due to the overlapping implant halation (arrowheads)

Furthermore, we assessed different measurement axes in addition to the axis used by Yoshino et al. [14]. One was defined by anatomical landmarks, and another defined by reference points determined on the implant itself. The position of the implant is commonly determined by how the bone is resected during surgery. Conventional intra- and extra-medullary guides determine the resection plane by setting the cutting block to a preoperatively planned angle against the rod, which is either inserted in the medullary canal or aligned outside the canal according to anatomical landmarks. Therefore, the precision of the surgery reflected by the postoperative alignment of TKA components, ideally should be evaluated by measuring the angle against the mechanical axis. However, we found it problematic to evaluate the traditional mechanical axis due to difficulty in identifying the anatomical landmarks on the postoperative CT, because of implant halation. It is possible to reduce implant halation on the image by image adjustment, but the bone contours also will be less clearly defined and become more difficult to identify (Fig. 4). Thus, we conducted the current study to evaluate measurement reliability using an axis defined by reference points set on the implant itself. Our results show that by minimizing the halation of the implant itself, the sharp edges of the component provide reference points to digitize and provide equivalent reliable and reproducible evaluation as anatomical landmarks. Thus, the implant-based axis may be utilized in situations in which the anatomical landmarks of the traditional mechanical axis are difficult to identify. Still, the slight difference between the two axes should be considered in interpretation of the results.

We obtained favorable intra- and inter-observer reliabilities using both traditional mechanical axis and implant-based axis in all but the intra-observer reliability of the sagittal alignment of the femoral component. This result was similar to the results obtained by Yoshino et al. [14] who also concluded that intra- and inter-observer reliabilities of the sagittal alignment of the femoral component were least reliable. Yoshino et al. considered the difficulty of image matching in this plane due to stronger blurring of the femoral component contour as the reason for the worse result [14]. In addition, it is conceivable that the reason for these poor results can be attributed to the round shape of the femoral component in the sagittal plane. Even with careful settings by referencing the pegs and sharp edges of the component, the round contour of the femoral component easily can cause an error of about 1–2º. In contrast, the edges of both femoral and tibial components are more easily visualized in the coronal plane, resulting in less error. The inter-observer ICC was calculated using the average of the two measurements made by each observer, resulting in a favorable value despite the poor intra-observer ICC in the measurement of sagittal alignment of the femoral component.

Adjusting the image acquisition protocol was one of the main concerns in designing this study. We tried to reduce the radiation by omitting the middle portion of the femur and tibia to reduce the area imaged, according to Henckel’s method [31]. However, the software we used required full limb CT data for accurate registration, and in a preliminary study, we experienced a number of cases in which the bone axis was not registered accurately. Thus, we concluded that a full leg CT was necessary for this study. The autoexposure control system was utilized to minimize radiation exposure [17].

This study has several limitations. First, the results we obtained cannot be generalized to all 3D-CT based systems as we did not compare the results from different platforms. However, it was not possible to test all systems. The system used in this study is a commonly used system in this region and the measurements were proved to be reliable and reproducible based on standard anatomical reference points. Thus, we believe that these results could be applied to other similar systems. Second, we did not assess the rotational alignment of the implanted components. The ability to evaluate the rotational alignment is a major advantage of 3D-CT imaging systems against two-dimensional imaging modalities. However, the primary purpose of this study was to analyze the accuracy of implant positioning in the coronal and sagittal planes. Furthermore, unlike the implant position in the coronal and sagittal planes, there is currently no standard reference axis to evaluate the rotational alignment of both femoral and tibial components after TKA [32]. Third, the results of this study may not be generalizable to all implants in that the traditional mechanical axis was slightly different from the implant-based axis. However, the main point at issue of this study was to investigate the reliability of component alignment assessment when measured by the reference point on the implant itself, considering occasions that bony landmarks are difficult to define on the CT images. From this point of view, we consider that the ICC of measurements would be similarly good for other implants if a reproducible reference point on the implant were defined. We conducted the study by taking the conceptual center of the components as reference points. While the center in the coronal plane will not vary much, the center of the component in the sagittal plane (antero-posterior center) could vary based on the definition of the reference points, resulting in variation of the flexion – extension alignment measurement. Thus, the degree of difference between traditional and implant-based axis measurements could vary between implants. However, as the “center of the component” would be expected to be located in a more or less similar position among different implants, we expect similar results to be obtained regardless of the type of implant.

## Conclusions

The postoperative evaluation of implant position in the coronal and sagittal planes using 3D-CT image matching is reliable with good reproducibility except for the sagittal alignment assessment of the femoral component. There was a small but significant difference between the measured implant position according to the traditional mechanical axis and the implant-based axis.

## Data Availability

The datasets used and/or analysed during the current study are available from the corresponding author on reasonable request.

## Abbreviations

TKA: Total knee arthroplasty
3D: Three-dimensional
CT: Computed tomography
CAD: Computer-aided-design
PS: Posterior-stabilized
CR: Cruciate-retaining
DICOM: Digital imaging and communications in medicine
ICC: Intraclass correlation coefficient
CI: Confidence interval
